# Validation of the Fatigue Severity Scale in chronic hepatitis C

**DOI:** 10.1186/1477-7525-12-90

**Published:** 2014-06-11

**Authors:** Kathleen Rosa, Min Fu, Leen Gilles, Karin Cerri, Monika Peeters, Jeffrey Bubb, Jane Scott

**Affiliations:** 1 University of North Carolina at Wilmington, Wilmington, NC, USA; 2 Janssen Research and Development, Radnor, PA, USA; 3 Janssen Research and Development, Beerse, Belgium; 4 Janssen Global Services, Global Commercial Strategy Organization, Beerse, Belgium; 5 Wu Consulting, Inc, Radnor, PA, USA; 6 Janssen Global Services, Global Commercial Strategic Organization, Manchester, UK

**Keywords:** Chronic hepatits C, Psychometric validation, Fatigue Severity Scale, Health status, Fatigue

## Abstract

**Background:**

Fatigue is a common symptom of chronic hepatitis C virus (cHCV) infection and a common side effect of interferon-based treatment for cHCV. This study provides confirmatory evidence of the reliability and validity of the Fatigue Severity Scale (FSS) to document fatigue in cHCV research and identifies values that indicate clinically important differences in FSS to aid in interpreting fatigue in cHCV clinical trials.

**Methods:**

The study used data from two double-blind, randomized, placebo-controlled, Phase IIb trials evaluating the efficacy and safety of simeprevir plus peginterferon-α/ribavirin in treatment-naïve (PILLAR, n = 386) and treatment-experienced patients (ASPIRE, n = 462) with cHCV infection. Patients completed the FSS and EuroQoL 5 dimension questionnaire (EQ-5D) at baseline and at regular intervals throughout both trials. Reliability was assessed using Cronbach’s coefficient α at Week 24 (internal consistency reliability) and intraclass correlation (ICC) between FSS at Weeks 12 and 24 in stable patients (<0.5 g/dL hemoglobin [Hb] change between Weeks 12/24). Correlation with the EQ-5D visual analog scale (VAS) and “Usual Activity” domain score was used to assess concurrent validity. Clinical validity was evaluated using a case-control method to link spontaneously reported fatigue and anemia adverse events (AEs) during the study to FSS scores.

**Results:**

FSS total scores demonstrated good reliability (Cronbach’s α: 0.95, 0.96; ICC: 0.74, 0.86 for PILLAR and ASPIRE, respectively) and concurrent validity (correlation with EQ-5D VAS: -0.63, -0.66) with a monotonic relationship between the EQ-5D “Usual Activities” item response and FSS. Clinical validity was confirmed by a significant difference between cases and controls for fatigue AEs (p < 0.05); however, anemia defined by AE or Hb abnormalities was only weakly related to FSS score. Analyses indicate that a change of 0.33–0.82 in mean FSS scores represents a meaningful improvement in fatigue, and a one-point change is a conservative indicator of an important change in individual FSS scores.

**Conclusion:**

A difference of ≥0.7 in mean FSS scores can be considered a clinically important difference within groups over time or between groups. A one-point change or less in individual FSS scores indicates a clinically relevant change in fatigue.

## Background

Fatigue is the most frequent symptom of chronic hepatitis C virus (cHCV) infection and also a common side effect associated with interferon-based therapies for cHCV infection [1-3]. Until recently, combination therapy with peginterferon-α and ribavirin (PegIFN-α/RBV) constituted the standard of care for HCV infection [4]. However, direct-acting antivirals (DAA) such as NS3/4A viral protease inhibitors when added to PegIFN-α/RBV have significantly improved the rate of sustained virologic response (SVR) rates compared with PegIFN-α/RBV alone [5-9]. Interferon-free treatments in development promise further reductions in treatment-induced fatigue [10].

The DAA simeprevir is a potent, once-daily, oral HCV NS3/4A protease inhibitor, recently approved for the treatment of cHCV infection. In two Phase IIb studies in treatment-naïve (PILLAR) and -experienced patients (ASPIRE) with cHCV infection, simeprevir in combination with PegIFN-α/RBV achieved significantly higher SVR rates compared with PegIFN-α/RBV alone [11,12]. In addition to efficacy and safety assessments, patients enrolled in the PILLAR and ASPIRE trials completed the Fatigue Severity Scale (FSS) in order to provide insight into the severity and duration of fatigue associated with the addition of simeprevir to PegIFN-α/RBV therapy. The FSS was selected to measure fatigue in the simeprevir trials because it is a brief, patient-reported outcome (PRO) measure of the impact of disabling fatigue on daily functioning in patients with chronic illness [13]. Initial validation of the FSS in patients with multiple sclerosis and systemic lupus erythematosus demonstrated good psychometric properties that were subsequently confirmed and extended in cross-cultural studies and in a variety of other chronic illnesses that have fatigue as a symptom or side effect of treatment [14,15]. When validated in patients with cHCV infection, the FSS produced a unidimensional construct with a resulting total score that was both valid and reliable [16]. The FSS has also been used to evaluate patients’ experience of fatigue in cHCV infection, both as a symptom of the disease and as an adverse event (AE) of treatment [17]. Lower levels of fatigue were reported in patients with SVR and an improvement in fatigue was associated with discontinuation of HCV therapy. What has yet to be determined is how to interpret the clinical significance of changes in FSS scores, either for evaluation of individual patients or group means in research [18]. Therefore, a key objective of this study was to establish empirical guides for interpreting FSS scores for HCV research.

Here we report the results of a psychometric analysis conducted to evaluate the adequacy and interpretation of FSS scores as a self-report measure of fatigue for clinical trials in patients with cHCV infection. The analysis used data from the PILLAR and ASPIRE trials in patients with cHCV infection treated with simeprevir or placebo in combination with PegIFN-α/RBV [11,12].

## Methods

### Data source

The PILLAR and ASPIRE studies were Phase IIb, multicenter, randomized, double-blind, placebo-controlled studies conducted in treatment-naïve (PILLAR, NCT00882908) or treatment-experienced (ASPIRE, NCT00980330) patients chronically infected with HCV [11,12]. The studies were reviewed and approved by the institutional review boards associated with each clinical site participating in the PILLAR and ASPIRE protocols. Each patient provided written informed consent prior to enrolling in the study. In the PILLAR study, patients were randomized to one of five treatments comprising simeprevir 75 or 150 mg/day for 12 or 24 weeks or placebo, plus PegIFN-α/RBV. Patients in the simeprevir arms could stop all treatment at Week 24 if HCV RNA testing indicated response to treatment based on response-guided therapy (RGT) criteria (HCV RNA <25 IU/mL at Week 4 and undetectable at Weeks 12, 16, and 20). Patients not meeting the RGT criteria continued with PegIFN-α/RBV until Week 48, as did all patients in the placebo control group (consistent with approved dosing for PegIFN-α/RBV treatment). In the ASPIRE study, patients with null or partial response or relapse to prior PegIFN-α/RBV received simeprevir 100 or 150 mg/day for 12, 24, or 48 weeks plus PegIFN-α/RBV for 48 weeks, or placebo plus PegIFN-α/RBV for 48 weeks. Follow-up was 72 weeks in both studies. An external physician reviewed HCV RNA levels throughout the blinded study to limit exposure to treatment for patients who experienced no viral response, viral breakthrough, or on-treatment relapse [11,12]. Patients and investigators remained blinded to the patient’s HCV RNA levels through Week 48 of treatment.

### Assessments

Patients completed the FSS and EuroQoL 5 dimension (EQ-5D) questionnaires at baseline, and at regular intervals during treatment and follow-up (FSS: Weeks 4, 12, 24, 36, 48, 60, 72; EQ-5D: Weeks 24, 48, 72). To minimize missing data for analysis, study personnel were instructed to review each completed questionnaire for missing responses and ask patients to complete any missing items before any other procedures were conducted during the study visit.

The FSS questionnaire instructs patients to assign a score of between 1 (completely disagree) and 7 (completely agree) to each of 9 FSS items designed to rate the extent of fatigue symptoms and their impact on patient functioning (including motivation, exercise, physical functioning, carrying out duties, and interfering with work, family, or social life). Examples of the questions asked include “exercise brings on my fatigue” and “my fatigue is very debilitating”; a higher score indicates a higher degree of fatigue for all items [18]. The standard scoring for the FSS was used: the 9 items were averaged to produce an FSS total score that ranges from 1 (no fatigue) to 7 (very severe fatigue) with scores calculated for all patients who answered at least half the FSS items. The FSS versions used in the PILLAR and ASPIRE studies differed in terms of length of recall period (i.e., patients were required to think back over a period of 14 days in PILLAR compared with 7 days in ASPIRE).

The EQ-5D is a self-administered questionnaire developed for measuring health status [19,20]. It comprises five questions relating to five health dimensions (Mobility, Self Care, Usual Activities, Pain/Discomfort, and Anxiety/Depression). Patients rate their current health status on each domain on a three level scale (level 1, no problems; level 2, some problems; level 3, extreme problems). The EQ-5D also includes a visual analog scale (VAS) which assesses overall health status on a scale of 0–100, with a score of 100 representing perfect health and a score of 0 the worst imaginable health status. For the FSS validation, two EQ-5D scores were used: the “Usual Activities” score from the EQ-5D descriptive system and the EQ-5D VAS.

During both studies, the investigators recorded the type, severity, and duration of any AEs spontaneously reported by patients. Fatigue and anemia AEs were identified using MedDRA preferred terms (“fatigue”, “aesthenia”, or “asthenia”; and “anaemia”, “haemoglobin decreased”, or “haemolytic anaemia”, respectively).

Blood samples were collected for determination of hemoglobin and plasma HCV RNA concentrations at baseline, Weeks 4 and 12, and then at 12-weekly intervals up to and including Week 72. For the purposes of the psychometric validation, patients were deemed no longer infected with HCV if they achieved an undetectable HCV RNA viral load at end of treatment (EOT) and at 24 weeks post treatment (SVR24).

### Analyses

Data from the PILLAR and ASPIRE studies were analyzed separately because the FSS recall periods were different for the two studies. Within each study, data were pooled across treatment groups. All analyses were conducted using SAS software (Statistical Analysis System, Version 9.2). The threshold for statistical significance was p < 0.05 for all comparisons.

#### ***Distribution***

The proportion of patients for whom the FSS total score could be calculated and the mean FSS total scores were determined. Percent of missing responses was calculated for each FSS item. Floor or ceiling effects at baseline were determined to assess the proportions of patients with the minimum possible (floor) or maximum possible (ceiling) values for individual FSS items and FSS total scores, and were considered present when more than 14.3% (calculated as 100/7, reflecting the 7 possible response categories for the FSS) of patients achieved these values. Analysis of floor and ceiling effects provides an indication of the ability of a PRO instrument to detect changes in a study population. If floor or ceiling effects are too pronounced, mean scores will be very high (ceiling) or very low (floor), making it difficult to detect changes over time.

#### ***Reliability***

Internal consistency reliability was assessed to evaluate the unidimensionality of the FSS total score, including the impact of item deletion. This was determined by calculating Cronbach’s coefficient α, with good internal consistency met if Cronbach’s α exceeded the widely accepted cut-off of 0.7 [21,22]. The impact of item removal on internal consistency reliability was examined by calculating Cronbach’s α after removal of each item from the total FSS score. An increase in Cronbach’s α after item deletion suggests that the item did not fit well with the total score.

Test-retest reliability that measures the degree to which an instrument produces similar scale scores at different points in time in “stable” patients was evaluated by calculating the intraclass correlation (ICC) [23]. The ICC was determined by comparing change in FSS total score at two different time points (Weeks 12 and 24) in a sub-group of stable patients, defined as individuals with <0.5 g/dL absolute change (improvement or worsening) in hemoglobin level between Weeks 12 and 24. An ICC value of at least 0.7 is considered evidence of acceptable test-retest reliability [24]. Pearson’s correlations were also calculated. The ICC is the preferred estimate of test-retest reliability over the Pearson’s correlation as it adjusts for any systematic mean shift that may be present between visits. If a mean shift is absent, the ICC and Pearson’s correlation are equivalent.

#### ***Clinical validity***

Known-groups validity assesses the extent to which scores are linked to a patient’s known health state or status based on clinical parameters that classify patients into groups. This was first evaluated by comparing FSS total scores and FSS change from baseline across four clinical sub-groups defined according to absolute hemoglobin level at EOT (normal, mild [Grade 1], moderate [Grade 2], severe/life-threatening [Grade 3/4]) [Table 1] [25].

**Table 1:** **Definition of clinical sub-groups by absolute hemoglobin level**[25]

**Hemoglobin**	**Female (g/dL)**	**Male (g/dL)**
Normal	>12	>13.5
Mild (Grade 1)	11-12	12.5-13.5
Moderate (Grade 2)	9.5-10.9	10.5-12.4
Severe/life-threatening (Grade 3/4)	<9.5	<10.5

Known-groups validity was also assessed based on SVR rate 24 weeks post treatment (SVR24) and on AE reports of anemia and fatigue. Both actual FSS total scores and change from baseline in FSS total score at the end of follow-up were compared for patients with and without SVR24 to evaluate the change in fatigue associated with achieving an undetectable HCV viral load. The first (or worst severity rated if more than one) fatigue or anemia AE that occurred during the FSS recall period (PILLAR: 2 weeks; ASPIRE 1 week) was recorded for each patient. For comparison purposes, a case-control method was employed whereby a control was selected from among patients who did not experience the relevant AE during the recall period, matching based on gender, age, and ethnicity. FSS scores at baseline and at the visit at which the worst AE occurred were reported, and change from baseline FSS scores were compared for cases versus controls.

Student t-tests were used for tests involving two groups, and one-way analysis of variance was used for tests involving three or more groups. Analyses were only performed for categories that included at least 20 patients in the smallest sub-group. Nonparametric methods were used if normality assumptions were not met.

#### ***Concurrent validity***

Concurrent validity is used to measure the correlation between two different PRO instruments that measure the same or related constructs. Using 24-week data, FSS total score was compared between groups, defined by response category for the EQ-5D “Usual Activities” item. A monotonic relation was pre-specified (i.e., lower FSS scores in patients with no problems versus some problems on the “Usual Activities” domain, and the highest scores in patients unable to perform “Usual Activities”). In addition, the correlation between FSS total score and overall health status, as measured by the EQ-5D VAS, was assessed based on 24-week data. A moderate and negative correlation was pre-specified based on the fact that a high VAS score represents good health and a high FSS total score indicates high fatigue levels.

#### ***Responsiveness***

Responsiveness measures the ability of an instrument to detect and measure change over time when underlying change has occurred in the construct being measured. In the planned preliminary analysis, clinically important changes in hemoglobin (1.0 g/dL change between visits) were proposed as a means of classifying patients as “improved” (at least a one-grade improvement in hemoglobin-defined anemia) or “worsened” (at least a one-grade change in hemoglobin-defined anemia). Different time points were used in the FSS responsiveness analyses to evaluate improvement (EOT versus end of study [EOS], i.e., Week 72) and worsening (baseline versus Week 24). This was necessary because the PILLAR and ASPIRE study protocols excluded patients with anemia at baseline (defined as hemoglobin <12 g/dL or <13.5 g/dL for females and males, respectively).

The second evaluation of responsiveness tested against the ability of the FSS to detect change in overall health status using the EQ-5D VAS, from baseline to Week 24 (“worsening”) and from EOT to EOS (“improvement”). Change groups were defined by a 10-point change on the VAS (reflecting 10% of the possible range for the VAS). Finally, responsiveness was evaluated using shift in the EQ-5D “Usual Activities” domain score from baseline to Week 24 (“worsening”) and from EOT to EOS (“improvement”).

#### ***Minimal important difference***

Minimal important difference (MID) provides a guide for interpreting the minimum change in a score. Distributional and anchor-based methods can be used for determining MID, with both producing generally similar results [26]. A distributional estimate of the MID was determined using the standard error of measurement (SEM) based on reliability of the FSS questionnaire (SEM = standard deviation [SD] × √[1-reliability]). The SEM is equivalent to ½ SD when reliability is equal to 0.75 and decreases as reliability increases. The ICC has been proposed as the most stable estimate to use in the calculation of the SEM [22]. For the anchor-based approach, the pre-specified analysis proposed a responder operating curve (ROC) analysis to establish the MID within-group change in FSS total score based on the changes in FSS scores that occur with clinically important changes in hemoglobin.

## Results

### Patients

A total of 386 treatment-naïve patients from the PILLAR study and 462 treatment-experienced patients from the ASPIRE study were included in the validation analysis. In both studies, the majority of patients (>90%) were Caucasian. Mean age was 44.0 years in the PILLAR study and 48.9 years in the ASPIRE study, and 55.2% and 67.3% of patients, respectively, were male [Table 2].

**Table 2:** Patient characteristics at baseline

	**Treatment-naïve (PILLAR)**		**Treatment-experienced (ASPIRE)**	
	**All patients (ITT)**	**Patients with baseline FSS and ≥1 follow-up**	**All patients (ITT)**	**Patients with baseline FSS and ≥1 follow-up**
	***N*** **= 386**	***N*** **= 241**	***N*** **= 462**	***N*** **= 446**
Sex, *n* (%)	*N* = 386	*N* = 241	*N* = 462	*N* = 446
Male	213 (55.2)	136 (56.4)	311 (67.3)	301 (67.5)
Female	173 (44.8)	105 (43.6)	151 (32.7)	145 (32.5)
Age, mean, SD (years)	44.0 (11.81)	45.9 (10.37)	48.9 (10.36)	48.9 (10.37)
Race, *n* (%)	*N* = 386	*N* = 241	*N* = 462	*N* = 446
Caucasian	362 (93.8)	225 (93.4)	428 (92.6)	414 (92.8)
Non-Caucasian	24 (6.2)	16 (6.6)	34 (7.4)	32 (7.2)
Region, *n* (%)	*N* = 386	*N* = 241	*N* = 462	*N* = 446
North America	82 (21.2)	52 (21.6)	120 (26.0)	113 (25.3)
Latin America	0	0	0	0
Europe/Israel	262 (67.9)	147 (61.0)	313 (67.7)	306 (68.6)
Asia	42 (10.9)	42 (17.4)	29 (6.3)	27 (6.1)
HCV RNA, mean (log_10_ IU/mL)	6.5 (0.61)	6.5 (0.64)	6.5 (0.57)	6.5 (0.56)
Genotype, *n* (%)	*N* = 383	*N* = 239	*N* = 455	*N* = 439
1a	173 (45.2)	122 (51.0)	188 (41.3)	178 (40.5)
1b	208 (54.3)	115 (48.1)	262 (57.6)	257 (58.5)
Other	2 (0.5)	2 (0.8)	5 (1.1)	4 (0.9)
METAVIR score, *n* (%)	*N* = 386	*N* = 241	N = 455	*N* = 439
F0–F2	332 (86.0)	203 (84.2)	286 (62.9)	278 (63.3)
F3	53 (13.7)	38 (15.8)	86 (18.9)	83 (18.9)
F4	1 (0.3)	0	83 (18.2)	78 (17.8)
Baseline PRO (mean)				
FSS total score	3.3 (1.58)	3.3 (1.57)	3.3 (1.65)	3.3 (1.65)
EQ-5D VAS	82.4 (15.28)	81.8 (15.75)	80.4 (16.47)	80.4 (16.42)

*EQ-5D VAS* European Quality of Life 5 dimension questionnaire visual analog scale, *FSS* Fatigue Severity Scale, *HCV* hepatitis C virus, *ITT* intent to treat, *PRO* patient-reported outcome, *SD* standard deviation.

### Baseline FSS item and total score distribution and floor-ceiling effects

**Figure 1:**
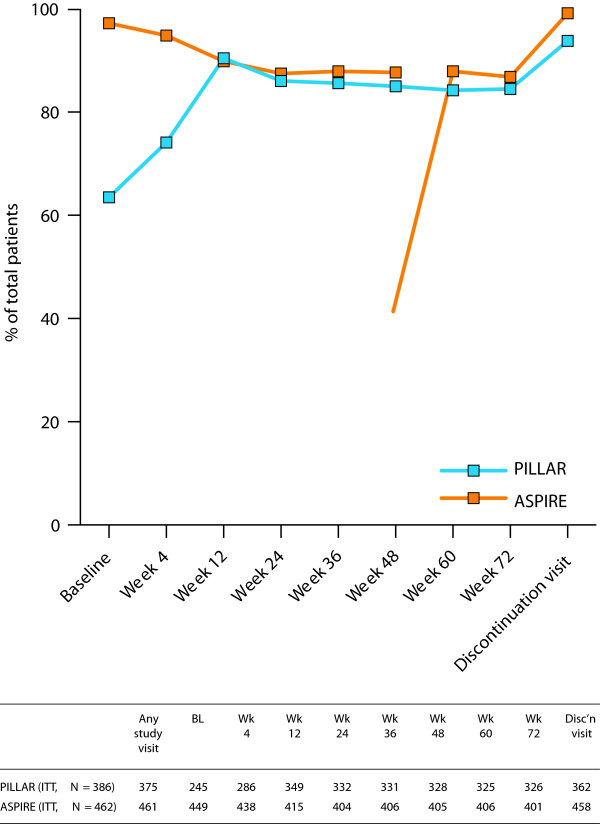
**Summary of available data for the FSS total score.** FSS total score was based on the mean of the 9-item score. If the number of missing items was <4, the FSS total score was the mean of the non-missing items; if 4 or more items were missing, the total score was set to missing. The PILLAR study was the first simeprevir trial to include PRO endpoints. Sites that enrolled the first patients did not have appropriate language translations of the PRO instruments for their patients to complete at their baseline visit, hence the low patient numbers at baseline in PILLAR (n = 245). Some of these patients subsequently completed PRO instruments at post-baseline visits. *BL* baseline, *FSS* Fatigue Severity Scale, *ITT* intent to treat, *PRO* patient-reported outcome.

The percentage of missing FSS item responses was low (≤0.4%) in both studies. A ceiling effect was observed for item 1 of the FSS (PILLAR 31%; ASPIRE 26%) indicating complete agreement with the statement “my motivation is lower when I’m fatigued”. The remaining 6 items had moderate to large floor effects (PILLAR 20.4-43.3%; ASPIRE 17.8-42.1%), endorsing the lowest response category, indicative of low levels of fatigue.Baseline FSS total scores were available for 245 of the 386 patients in the PILLAR study (mean [SD]: 3.28 [1.58]) and for 449 of the 462 patients (mean [SD]: 3.34 [1.65]) in the ASPIRE study [Figure 1]. Only 4.9% of patients had the lowest possible score (floor) and 1.2% had the highest possible score (ceiling) in the PILLAR study. The corresponding results for the ASPIRE study were 8.0% (floor) and 2.7% (ceiling). Together, these findings confirmed that the floor and ceiling effects were of no concern in either study.

### Internal consistency

Cronbach’s coefficient α was above the cut-off of 0.7 for FSS at baseline, indicating high internal consistency in both studies (PILLAR 0.96; ASPIRE 0.96) [Table 3]. For all except the first FSS item, Cronbach’s α decreased slightly after deletion of each item, confirming that the items contribute to the total score and belong to the scale. The fact that deletion of the first item did not reduce Cronbach’s α suggests that it does not contribute to the FSS total score.

**Table 3:** Reliability of the FSS

**FSS scores**	**Treatment-naïve (PILLAR)**	**Treatment-experienced (ASPIRE)**
Test-retest reliability^a^		
ICC	0.74	0.86
Pearson’s correlation	0.74	0.86
Internal consistency		
Cronbach’s α	0.95	0.96

*FSS* Fatigue Severity Scale, *ICC* intraclass correlation.

^a^Test-retest reliability was calculated using data from Week 12 and Week 24, using only stable patients defined by a change in hemoglobin of <0.5 g/dL.

### Test-retest reliability

ICC values for the PILLAR and ASPIRE studies (0.74 and 0.86, respectively) met the established criteria for test-retest reliability. Results also indicated no mean shift (ICC compared with Pearson’s correlation) in FSS total score [Table 3]. A total of 159 patients in the PILLAR study and 147 patients in the ASPIRE study were categorized as stable. Mean (SD) total FSS score at Weeks 12 and 24 were very similar (PILLAR: 4.3 [1.49] and 4.3 [1.56], respectively, p = 0.652; ASPIRE: 4.2 [1.70] and 4.2 [1.68], respectively, p = 0.635).

### Clinical validity

In both the PILLAR and ASPIRE studies, patients with normal hemoglobin levels or mildly suppressed hemoglobin at EOT had similar mean FSS total scores, while patients with abnormal hemoglobin, indicating moderate to severe anemia, had higher (worse) fatigue scores [Table 4]. Change in FSS score from baseline increased as expected as hemoglobin decreased, achieving statistical significance (moderate/severe versus normal, p = 0.014) in the PILLAR study but showed minimal change between hemoglobin categories in the ASPIRE study.

**Table 4:** Validity of the FSS

**FSS scores**	**Actual score at time of clinical outcome**		**Change from baseline to clinical event**			
	**Treatment-naïve**	**Treatment-experienced**	**Treatment-naïve**	**P value**	**Treatment-experienced**	**P value**
	**(PILLAR)**	**(ASPIRE)**	**(PILLAR)**		**(ASPIRE)**	
	**Mean (SD)**	**Mean (SD)**	**Mean CFB (SD)**		**Mean CFB (SD)**	
SVR24						
Yes	2.7 (1.59)	3.0 (1.63)	-0.6 (1.41)		-0.4 (1.42)	
No	3.5 (1.83)	3.6 (1.80)	0.3 (1.28)	***	0.4 (1.50)	***
Hemoglobin (EOT)^a^						
Normal	4.3 (1.76)	4.3 (1.77)	0.8 (1.54)	(comparator)	1.0 (1.56)	(comparator)
Mild anemia	4.3 (1.67)	4.2 (1.73)	1.2 (1.67)	n.s.	0.9 (1.53)	n.s.
Moderate/severe anemia	4.7 (1.55)	4.5 (1.73)	1.5 (1.56)	*	1.1 (1.62)	n.s.
Anemia AE						
Case	4.5 (1.79)	4.8 (1.70)	1.2 (1.76)		1.1 (1.72)	
Control	4.2 (1.73)	4.4 (1.74)	1.0 (1.38)	n.s.	1.0 (1.38)	n.s.
Fatigue AE						
Case	4.4 (1.58)	4.5 (1.59)	1.1 (1.45)		0.9 (1.35)	
Control	3.8 (1.67)	3.5 (1.67)	0.5 (1.49)	*	0.4 (1.26)	***
EQ-5D “Usual Activities”						
“No problems”	3.3 (1.31)	3.4 (1.51)	0.6 (1.29)	(comparator)	0.6 (1.38)	(comparator)
“Some problems”	5.2 (1.23)	5.4 (1.19)	1.6 (1.64)	***	1.5 (1.45)	***
“Cannot perform”^b^	6.5 (0.48)	6.5 (1.15)	2.2 (1.97)	**	1.4 (1.69)	*

*AE* adverse event, *CFB* change from baseline, *EOT* end of treatment, *EQ-5D* European Quality of Life 5 dimension questionnaire, *FSS*, Fatigue Severity Scale, *n.s.* not significant, *SD* standard deviation, *SVR24* sustained virologic response rate 24 weeks post treatment.

*p < 0.05; **p < 0.01; ***p < 0.001.

^a^Clinical groups were defined as normal: >12 g/dL (female), >13.5 g/dL (male); mild (Grade 1) 11–12 g/dL (female), 12.5–13.5 g/dL (male); moderate (Grade 2) 9.5–10.9 g/dL (female), 10.5–12.4 g/dL (male); severe/life-threatening (Grade 3/4) <9.5 g/dL (female), <10.5 g/dL (male).

^b^The “cannot perform” group included low numbers of patients: PILLAR, n = 8; ASPIRE, n = 16.

**Figure 2:**
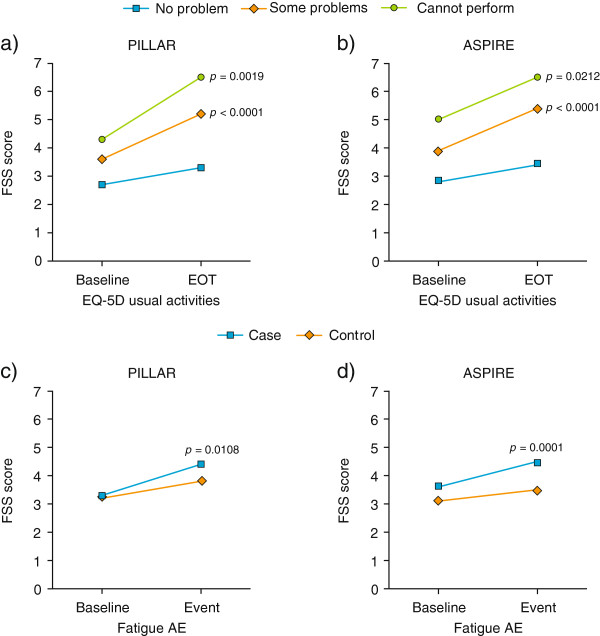
**Validity of the FSS.** Mean change from baseline at EOT or event; **a)** and **b)** patients with no problems, some problems or patients who could not perform the EQ-5D “Usual Activities” dimension at EOT; **c)** and **d)** patients (cases) with fatigue AEs versus control patients. p values versus “no problem” (a and b) or versus “control” (c and d). *AE* adverse event, *EOT* end of treatment, *EQ-5D* European Quality of Life 5 dimension questionnaire, *FSS* Fatigue Severity Score.

Patients with fatigue AEs had consistently higher FSS scores than control patients in both the PILLAR and ASPIRE studies [Table 4; Figure 2c and d]. Although both the case and control patients experienced a worsening of their total FSS score from baseline, the deterioration was more pronounced among patients with fatigue versus controls and reached statistical significance in both studies. FSS total scores were not significantly different for patients with and without anemia AEs.

Patients who achieved SVR24 had lower mean FSS total scores compared with patients who did not in both the PILLAR and ASPIRE studies [Table 4]. Mean change from baseline FSS total scores indicated that patients with SVR24 improved by 0.6 (PILLAR) or 0.4 points (ASPIRE) while patients without SVR worsened by 0.3 and 0.4 points, respectively (p < 0.001 for between-group comparisons in both studies).

### Concurrent validity

In both the PILLAR and ASPIRE studies, patients grouped by responses to the EQ-5D “Usual Activities” item at Week 24 showed the predicted monotonic relation with total FSS score [Table 4]. Patients with “no problems” performing “Usual Activities” had lower mean FSS total scores than patients with “some problems”; patients in the “cannot perform usual activities” category had the highest FSS scores indicating the most severe fatigue as expected [Figure 2a and b]. Compared with the “no problems” group, the differences in change from baseline FSS scores were significant for the “some problems” group (p < 0.001 both studies) and the “cannot perform” group in both studies (PILLAR, p = 0.002; ASPIRE, p = 0.021). Of note, change from baseline in FSS total score was monotonic in the PILLAR study but not in the ASPIRE study; this may have been attributable to high baseline FSS total scores in the “cannot perform” group in the ASPIRE study, possibly reflecting the greater proportion of patients in ASPIRE with advanced liver fibrosis. The correlation between the FSS total score and EQ-5D VAS was moderate and in the direction expected (PILLAR, r = -0.63; ASPIRE, r = -0.66).

### Responsiveness

The FSS showed good responsiveness as summarized in Table 5; however, responsiveness to changes in anemia produced weak results. Patients who “worsened” based on hemoglobin abnormalities used to define anemia (Grade 1 - 4) showed a greater mean increase in FSS total score from baseline to Week 24 (denoting worsened fatigue) compared with the “not worsened” group in both studies. The differences between groups in both studies failed to reach statistical significance using parametric testing, although statistical significance was achieved using the Wilcoxon rank-sum test in the PILLAR study (p < 0.05). Improvement in FSS total score from EOT to EOS was significantly greater in the “improved” patient group (at least a one-category improvement in hemoglobin) than in the “unimproved group” (p = 0.006) in the PILLAR study and was greater, albeit not statistically significant, in the “improved” versus “unimproved” group in the ASPIRE study.

**Table 5:** Responsiveness of the FSS score

**FSS scores**	**Treatment-naïve**		**Treatment-experienced**	
	**(PILLAR)**		**(ASPIRE)**	
	**Mean CFB (SD)**	**P value**	**Mean CFB (SD)**	**P value**
Hemoglobin				
*Baseline to Week 24*^a^				
Worsened	1.3 (1.61)		1.0 (1.47)	
Not worsened	0.8 (1.48)	n.s.	0.8 (1.51)	n.s.
*EOT to 24-wk FU*^*b*^				
Improved	-1.6 (1.73)		-1.3 (1.55)	
Unimproved	-1.1 (1.38)	**	-1.0 (1.52)	n.s.
EQ-5D VAS				
*Baseline to Week 24*				
Worsened	1.6 (1.61)		1.5 (1.47)	
Not worsened	0.5 (1.33)	***	0.5 (1.33)	***
*EOT to 24-wk FU*				
Improved	-2.1 (1.62)		-1.6 (1.65)	
Unimproved	-0.8 (1.37)	***	-0.7 (1.23)	***
EQ-5D “Usual Activities”				
*Baseline to Week 24*				
“No problems” to “some problems”	1.90 (1.51)		1.72 (1.44)	
Not worsened	0.52 (1.35)	***	0.57 (1.34)	***
*EOT to 24-wk FU*				
“Some problems” to “no problems”	-2.27 (1.68)		-2.05 (1.53)	
Unimproved	-0.98 (1.30)	***	-0.80 (1.35)	***

*CFB* change from baseline, *EOS* end of study, *EOT* end of treatment, *EQ-5D VAS*, European Quality of Life 5 dimension questionnaire visual analog scale, *FSS* Fatigue Severity Scale, *FU* follow-up, *n.s.* not significant, *SD* standard deviation.

**p < 0.01; ***p < 0.001.

^a^“Worsened” was defined, using data from baseline and Week 24, as those patients with at least a Grade 1 change in anemia based on hemoglobin levels.

^b^“Improved” was defined, using data from EOT and EOT + 24 weeks (EOS, 72 weeks), as at least a Grade 1 improvement in anemia based on hemoglobin levels.

In the responsiveness assessment based on important change in the EQ-5D VAS, mean change from baseline to Week 24 in FSS total score was significantly worse in the “worsened” group (≥10-point worsening on the EQ-5D VAS) compared with the “not worsened” group (<10-point improvement/worsening on the EQ-5D VAS) (p < 0.001 for both studies). Similarly, improvement in FSS total score from EOT to EOS in the “improved” group (≥10-point improvement in EQ-5D VAS) was significantly greater than that observed in the “unimproved” group (p < 0.001 for both studies) [Table 5].

Finally, in the responsiveness assessment based on groups defined by category shift in the EQ-5D “Usual Activities” response, mean change from baseline to Week 24 in FSS total score was significantly worse in the “worsened” group (patients who shifted from “no problems” to “some problems”) compared with the “not worsened” group (p < 0.001 for both studies). Similarly, improvement in FSS total score from EOT to EOS in the “improved” group (patients who shifted from “some problems” to “no problems”) was significantly greater than that observed in the “unimproved” group (p < 0.001 for both studies) [Table 5].

### Minimal important difference

Due to inadequate numbers of patients with the defined point changes in hemoglobin, the planned ROC analysis was not performed. Distribution-based estimates of the MID based on the PILLAR study suggested that an observed group mean change in FSS score of between 0.34 and 0.79 was indicative of an interpretable and meaningful improvement in fatigue. Similar findings were reported for the ASPIRE study (mean change 0.33 - 0.82).

## Discussion

This analysis used data from two randomized, double-blind, placebo-controlled studies conducted in treatment-naïve and -experienced patients with cHCV infection to reconfirm that the FSS is a valid, reliable, and responsive measure of fatigue for use in HCV treatment trials. An important and unique aspect of this study was the identification of the degree of change in the FSS that can be considered clinically meaningful.

At baseline, a floor effect was observed for 6 of the 7 FSS items. Although the PILLAR and ASPIRE studies enrolled patients who potentially experienced some fatigue at baseline as a result of cHCV infection, much of the fatigue observed in these trials was attributable to side effects of treatment. Therefore, it was not unreasonable for large floor effects, indicative of low levels of fatigue, to exist at baseline. Large floor effects would not interfere with the measurement goal of evaluating increased fatigue but could make it difficult to evaluate improvements in fatigue, potentially attenuating any observed benefit of SVR on fatigue. Floor–ceiling effects for the FSS total score were minimal.

Internal consistency reliability of the FSS was very good. The item level deletion results suggested that the first FSS item (“my motivation is lower when I am fatigued”) did not add consistent information and could potentially be removed from the scale for future work. This finding was not unexpected given that the wording of item 1 is different from that of the other items, being more general rather than directly evaluating the patient’s current state of fatigue. Test-retest reliability surpassed the pre-specified criteria of 0.70 for both studies. As predicted, the FSS had moderate negative correlations with the EQ-5D VAS and showed a monotonic relationship with the EQ-5D “Usual Activities” item, with results consistent between the two studies.

Results from clinical validity analyses relating the FSS to hemoglobin levels were mixed. In general, total FSS scores were similar in patients with normal or mild hemoglobin abnormalities. This is consistent with current descriptions of mild anemia as symptom free [27,28]. Patients with moderate or high (at least Grade 2) hemoglobin levels differed from patients without significantly reduced hemoglobin levels, with a highly significant effect in the PILLAR study and a moderate, non-significant effect in the ASPIRE study.

Reports of anemia AEs were only weakly related to FSS. There was a non-significant trend towards a greater change in total FSS score from baseline to the recall period for the reported event for patients with anemia AEs compared with controls in both studies. In contrast, there was a strong association between reports of fatigue AEs and FSS, with change in total FSS score from baseline to the recall period for the event significantly higher for cases versus controls in both studies.

The FSS also had a strong relation with SVR in both studies, with patients achieving SVR at study end having significantly lower FSS total scores than patients without SVR. Our findings are consistent with the recommendations provided by Sarkar et al. in their report of the fatigue results from the Virahep-C study [2]. Based on a simple VAS measure of fatigue collected before, during, and after treatment for HCV, the authors reported that the presence and severity of fatigue ultimately declined in patients with sustained clearance of HCV. They commented that their findings indicated that HCV therapy can lead to significant and sustained improvement in clinical symptoms, and that the measurement of fatigue using VAS is successful in capturing these changes, with improvements in fatigue being most convincing in those patients with moderate to severe levels of fatigue at baseline. The authors concluded that those patients with relatively nonsignificant biochemical or histologic disease, but with troublesome symptoms such as fatigue, should be considered for antiviral therapy [2].

The responsiveness analysis indicated that the FSS score measures change where change in fatigue-related concepts exists; however, responsiveness to changes in anemia produced weak results. While we expected a relationship between self-reported fatigue and anemia AEs, other causes for fatigue have been suggested in the literature, including increased HCV viremia in the brain and central nervous system [29]. However, at the time of writing no studies have definitively linked central nervous system levels of HCV viremia with patient-reported fatigue. A multivariate approach could potentially uncover the interactive relationship of anemia as a treatment-emergent AE and SVR in predicting fatigue, and may be a fruitful area of future research.

In addition to understanding whether a PRO instrument can detect change, it is also essential to understand the meaning associated with the change. The point at which a change score becomes important has been characterized as the MID. The MID is often misunderstood to represent the point difference that must be present between treatment groups in order for a difference to be considered clinically meaningful. Instead, the MID should be considered to be a within-group phenomenon [30] and any between-group effect size should actually be smaller than the MID [31]. During the design of a study, clinical knowledge of the indication and the expected effect of both treatment and control should guide estimates of effect size. For example, one might expect a very small effect between a treatment and an active comparator, and a larger effect between placebo and treatment. The accurate estimation of sample size to test a certain effect size takes into account the precision of the endpoint. On this basis, the MID should be used to guide decisions about change within a group that can be considered meaningful. A clinically meaningful between-groups effect size is not an artifact of the instrument in use, but instead should be determined based upon clinical and statistical considerations. As indicated in the FDA’s PRO Guidance [32], these methods are not appropriate for determining the precise point at which a score change indicates clinical significance but are useful for evaluating the meaning of an observed score change.

The MID or the meaning associated with changes in FSS scores was assessed using distributional estimates. Results from both studies suggest that an interpretable and meaningful improvement in fatigue occurs when there is an observed-group mean change in FSS total score of between 0.33 and 0.82. Although the planned anchor-based analyses could not be performed because of insufficient sample with significantly suppressed hemoglobin levels, additional information on the importance of change scores can be gained through evaluation of the analyses involving the EQ-5D and fatigue AE events as previously discussed. Considered together, the results support a one-point change in an individual’s FSS score as a reasonable and appropriate responder definition. These results have been confirmed in a pooled analysis of three simeprevir Phase III clinical trials [33].

Together these results extend our understanding of the utility and interpretation of the FSS for research in patients with cHCV. The earlier study by Kleinman et al. used screening and baseline visits to evaluate reliability and convergent validity of the FSS with other outcomes [18]. Our study adds to this by showing the link between FSS scores and clinicians’ ratings for fatigue and anemia, but also hemoglobin abnormalities and SVR. The current study provides guides that can be used to evaluate group means or individual scores that have been confirmed using data from over 800 patients, including treatment-naïve and treatment-experienced patients, during treatment for cHCV. Future studies using the FSS can better estimate the statistical power required for evaluating fatigue effects given these findings. Our findings suggest that the FSS may also be used to evaluate whether fatigue levels warrant initiation of HCV treatment and to ensure treatment-emergent fatigue is detected and managed successfully.

## Conclusion

These analyses indicate the FSS is a valid and reliable PRO tool appropriate for use in clinical trials involving patients with HCV infection. The current results support a responder definition of one point on the FSS score; however, cumulative response distributions at critical time points are recommended to aid interpretation of parametric results. Further work is required to understand the relationship between fatigue, anemia, and hemoglobin abnormalities, particularly in treatment-experienced HCV patients undergoing retreatment; this will likely require inclusion of a large patient population and a multivariate approach to separate out disease effects, positive response to treatment, and adverse effects of treatment.

## Abbreviations

AE: Adverse event; BL: Baseline; CFB: Change from baseline; cHCV: Chronic hepatitis C virus; EOS: End of study; EOT: End of treatment; EQ-5D: EuroQoL 5 dimension questionnaire; EuroQoL: European Quality of Life; FSS: Fatigue Severity Scale; Hb: Hemoglobin; ICC: Intraclass correlation; ITT: Intent to treat; MID: Minimal important difference; n.s.: not significant; PegIFN-α: Peginterferon-α; PRO: Patient-reported outcome; RBV: Ribavirin; RGT: Response-guided therapy; SD: Standard deviation; SEM: Standard error of measurement; SVR: Sustained virologic response; SVR24: SVR rate 24 weeks post treatment; VAS: Visual analog scale.

## Competing interests

Kathleen Rosa is a paid consultant to Janssen and owns no Janssen stock.

Jeffrey Bubb owns stock in Johnson & Johnson.

Min Fu, Leen Gilles, Karin Cerri, Monika Peeters, and Jane Scott are employees of Janssen and own Janssen stock.

## Authors’ contributions

KR contributed to the conceptualization of the study, planning of the analyses, writing of the analysis plan and final report for the study, and writing and reviewing of the manuscript. JS led the development and conceptualization of the study and the drafting and final manuscript reporting this research. JB programmed the output referenced in the tables. LG was primary statistician for the analysis of the PRO measures in PILLAR and ASPIRE. MF provided statistical oversight for psychometric analyses. MP was the biostatistics leader for the PILLAR and ASPIRE trials. All authors provided comments on the analysis results reported here and approved the final version of this manuscript.

## Authors’ information

Kathleen Rosa is a psychometrician with a PhD in Quantitative Psychology from UNC Chapel Hill. She has worked in the planning and analysis of clinical trials for over 15 years, with a specialization in developing, validating, and implementing PROs for clinical trial endpoints.
